# Exploring the beliefs, experiences and impacts of HIV-related self-stigma amongst adolescents and young adults living with HIV in Harare, Zimbabwe: A qualitative study

**DOI:** 10.1371/journal.pone.0268498

**Published:** 2022-05-18

**Authors:** Camille Rich, Webster Mavhu, Nadine Ferris France, Vongai Munatsi, Elaine Byrne, Nicola Willis, Ann Nolan

**Affiliations:** 1 Trinity Centre for Global Health, Trinity College Dublin, the University of Dublin, Dublin, Ireland; 2 Centre for Sexual Health & HIV/AIDS Research (CeSHHAR), Harare, Zimbabwe; 3 Liverpool School of Tropical Medicine, Liverpool, United Kingdom; 4 Beyond Stigma, Dublin, Ireland; 5 The Irish Global Health Network, Dublin, Ireland; 6 Africaid, Harare, Zimbabwe; 7 Institute of Leadership, Royal College of Surgeons in Ireland (RCSI), University of Medicine and Health Sciences, Dublin, Ireland; Brown University, UNITED STATES

## Abstract

**Background:**

HIV-related self-stigma is a significant barrier to HIV management. However, very little research has explored this phenomenon, particularly in sub-Saharan Africa. This study explored the beliefs, experiences, and impacts of HIV self-stigma amongst adolescents and young adults (AYALHIV) in Harare, Zimbabwe to inform future interventions. It aimed to capture the lived experience of self-stigmatization among AYALHIV and its impact on their social context using Corrigan et al (2009) self-stigma framework of ‘awareness’, ‘agreement’, and ‘application’.

**Methods:**

Virtual semi-structured key informant interviews were conducted between June and July 2020 with adolescents and young adults (Female = 8; Male = 8) living with HIV (18–24 years) in Harare, Zimbabwe. We conducted the interviews with a purposive sample of AYALHIV enrolled in Africaid’s ‘Zvandiri’ program which provides HIV support services. Interviews were mainly conducted in English and with three in Shona, the main indigenous language. Audio-recorded qualitative data were transcribed, translated into English (where necessary) and deductively coded using Corrigan et al.’s self-stigma framework. The outbreak of SARS-CoV-2 coincided with the commencement of data collection activities, which impacted on both the sample size and a shift from in-person to virtual interviewing methods.

**Results:**

Sixteen respondents (50% male) took part in the interviews. The mean age of respondents was 22 years. All respondents reported HIV-related self-stigma either occasionally or frequently. Three main themes of self-stigmatizing experiences emerged: disclosure, relationships, and isolation. These themes were then analyzed within the self-stigma development framework by Corrigan et al. (2009) known as ‘the three As’: awareness, agreement, and application of self-stigmatizing thoughts. Respondents’ experiences of self-stigma reportedly led to poor well-being and decreased mental and physical health. Gendered experiences and coping mechanisms of self-stigma were reported. Data suggested that context is key in the way that HIV is understood and how it then impacts the way people living with HIV (PLHIV) live with, and experience, HIV.

**Conclusions:**

HIV-related negative self-perceptions were described by all respondents in this study, associated with self-stigmatizing beliefs that adversely affected respondents’ quality of life. Study findings supported Corrigan et al.’s framework on how to identify self-stigma and was a useful lens through which to understand HIV-related self-stigma among young people in Harare. Study findings highlight the need for interventions targeting PLHIV and AYALHIV to be context relevant if they are to build individual resilience, while working concurrently with socio-political and systemic approaches that challenge attitudes to HIV at the wider societal levels. Finally, the gendered experiences of self-stigma point to the intersecting layers of self-stigma that are likely to be felt by particularly marginalized populations living with HIV and should be further explored.

## Introduction

Stigma is a significant barrier to HIV treatment and management [1, 2]. HIV remains a highly stigmatized condition because of what are considered high risk or taboo behaviors associated with its transmission, such as multiple and concurrent sexual partnerships, homosexual intercourse, drug use and sex work [3–6]. HIV-related stigma can be experienced as social or internalized self-stigma [7]. Social stigma is the outward discrimination towards people living with HIV (PLHIV) [8]. Self-stigma on the other hand, is experiencing negative judgements towards oneself resulting in feelings of worthlessness, shame and emotional distress [9]. For example, a self-stigmatizing thought is, ‘I will never have a future because I am worthless and not good enough.’ There is little research on self-stigma and how it impacts different population groups living with HIV [10]. As a result, this study focuses on the beliefs and experiences of HIV self-stigma amongst young adults living with HIV in Harare, Zimbabwe, an area deeply affected by the HIV/AIDS epidemic.

Corrigan et al. (2009) identify three steps of self-stigmatization [11]. First, one has to have an awareness of a stereotype or negative perception. Then one has to agree with those beliefs, and finally, apply them onto one’s self [11]. This process is described as the three A’s of self-stigmatization, which are awareness, agreement, and application [11].

While social stigma has been the primary focus of HIV stigma research, much less is known about HIV-related self-stigma [10]. Studies have suggested that HIV-related self-stigma can have detrimental physical and mental effects on PLHIV [12]. Research has shown that HIV-related self-stigma can result in non-adherence to HIV medication, lower quality of life and poor mental health [13, 14]. A systematic review found that HIV-related stigma undermined antiretroviral therapy (ART) adherence by compromising general psychological processes (e.g. adaptive coping and social support), and 24/33 (73%) studies reported an association between HIV stigma and ART non-adherence [15].

Researchers found that self-stigma can be experienced almost three times more than social stigma, demonstrating a need to both better understand and address this phenomenon [16]. Data suggest that self-stigma among PLHIV intersects with age, gender and personal experiences [17].

### Self-stigma within Zimbabwean/sub-Saharan context

Over 1.4 million people are living with HIV in Zimbabwe and the adult prevalence rate of HIV is 12.7% [18, 19]. Young adults have been identified as a critical population affected by HIV and one third of all new infections in Zimbabwe are currently among people aged 15–24 years old [18, 19]. These young PLHIV can internalize negative stereotypes associated with HIV and experience feelings of isolation, negative self-image, worthlessness and suicidal thoughts [12].

Previous research conducted in Zimbabwe showed that adolescents living with HIV (ALHIV) in Zimbabwe are particularly affected by stigma and self-stigma [20–22]. This report stated that 73% of ALHIV experienced stigma that affected their medication adherence and 47% had virological failure [22]. The stigma experiences reported included abuse, negative self-perception, neglect, social isolation, and discrimination [22]. Additionally, some of the indigenous terms used to identify PLHIV include derogatory terms that translate into “dead one” or “plagued” [23]. This linguistic phenomenon reinforces an unfavorable and hopeless view of HIV [23].

Self-stigma contributes to high drop-out rates from HIV treatment and support programs among young people, leading to increased morbidity and mortality [22, 24, 25]. Self-stigma can cause PLHIV to refrain from HIV testing, default on their medication, avoid going to health clinics and experience mental health problems, which increases negative health outcomes [26]. It is particularly critical to address HIV-related self-stigma in sub-Saharan Africa, a region home to 85% of the world’s adolescents living with HIV and the only population with increasing mortality rates [24, 27, 28].

### Study focus and rationale

This research explored beliefs, experiences and impacts of HIV-related self-stigma amongst older adolescents and young adults (18–24 years) in Harare, a group with disproportionately poor HIV clinical and psychosocial outcomes [25, 27, 29]. It aimed to understand the experience of self-stigmatization within this population and context through Corrigan’s framework of self-stigma: awareness, agreement, and application. This understanding will help inform self-stigma reduction interventions in Zimbabwe and sub-Saharan Africa more widely. Addressing HIV-related stigma in general and self-stigma in particular, is important for improving AYALHIV’s clinical and psychosocial outcomes [12].

## Methods

### Design and setting

We conducted this qualitative study with adolescents and young adults living with HIV (AYALHIV) (18–24 years) enrolled in Africaid’s Zvandiri program, in Harare, Zimbabwe. The Zvandiri (meaning ’As I am’) program provides services, counselling, medication, and support to children, adolescents, and young people living with HIV in Zimbabwe and eight other countries [22, 30]. Zvandiri aims to directly improve the well-being of this population thereby strengthening their engagement with services across the HIV prevention and care cascades [22, 30]. This study used both convenience and purposive sampling to identify respondents as all were participants in the Zvandiri programme and living, as young adults, with HIV. Sixteen respondents were interviewed; 12 (75%) were trained peer supporters known as Community Adolescent Treatment Supporters (CATS) and 4 were beneficiaries (a term used in Zvandiri for those clients who are not CATS). Purposive sampling guided an equal selection of females (n = 8) and males (n = 8). Interviews were conducted between June and July 2020. The outbreak of SARS-CoV-2 coincided with the commencement of data collection activities, which impacted on both the sample size and a shift from in-person to virtual interviewing methods.

### Theoretical paradigm and positionality

Deductive reasoning using Corrigan et al’s (2009) self-stigma framework [11] informed the development of the interview guide (see S1 File). Questions were grouped by the three A’s of self-stigma: awareness, agreement, and application in order to efficiently analyze the potential experiences of self-stigma [11]. The interview guide was designed as a flexible semi-structured guide in order to facilitate the emergence of respondents’ lived experiences. Therefore, this research was both deductive and inductive. Deductive analysis was used with the self-stigma framework to determine if respondents actually experienced HIV-related self-stigma. The discussions with the respondents were compared to Corrigan et al’s (2009) self-stigma framework [11] in order to have consistency as to what is considered the experience of self-stigma.

However, an inductive and phenomenological approach was also used to determine the themes within this framework of the beliefs and experiences of HIV-related self-stigma as experienced by the respondents. This inductive approach is rooted in the sociological theory of social constructivism; which understands human experiences as socially created and shaped by a person’s engagement with their culture and society [31]. This paradigm describes reality as unique for different individuals based on how they make sense of the world through a combination of history, social norms, and community interactions [32]. The social constructivist approach interprets the phenomenon of HIV-related self-stigma from the perspective of the respondents and their unique context which helped to develop the sub themes within Corrigan et al’s (2009) three A’s of self-stigma: awareness, agreement, and application [11, 32].

As adult researchers, we also employed self-critical epistemological awareness considering how our own perceptions and experiences could potentially influence interpretation of study findings. Out team included Zimbabwean researchers for local and in-depth context, as well as non-Zimbabwean self-stigma experts. We privileged the voices of study respondents and ensured we set aside our own biases to maximise rigour during data analysis, interpretation, and reporting (e.g., through researcher triangulation).

### Data collection, processing and analysis

All interviews were led by CR in English in the presence of Africaid’s psychologist (VM) who was known to all research respondents. While the psychologist presence may have had some impact on what the respondents revealed, the familiar support provided by the psychologist ensured the safety and security of respondents and provided immediate emotional support if required. Additionally, the psychologist acted as a live translator in the event that any of the respondents needed to respond in Shona. Any translations were time stamped and recorded in the data.

Due to the COVID-19 pandemic, interviews were conducted virtually through the Zoom video conferencing platform because the lead researcher was unable to travel to Harare. With the support of a travel stipend, respondents came to the Africaid facility and participated in the interviews in a private space. Each interview lasted 30–45 minutes and was recorded with respondents’ permission. The recorded interviews were transcribed (and the sections in Shona were translated) verbatim into English.

A summary of the discussion was written following each interview. These summaries and weekly analytical meetings amongst the research team informed development of a provisional coding framework. Half of the interviews (n = 8) were then independently coded by two researchers (CR,WM) using Corrigan et al’s (2009) framework [11]. Discrepancies were resolved through discussion (with EB) until consensus was reached. Any additional codes identified from this first set of transcripts were added to the coding framework. Transcripts were entered into NVivo 12 (QSR International, Melbourne, Australia) and fully coded using the modified coding framework; care was taken to identify any additional emerging codes. Codes were grouped and emerging themes were identified and supported with verbatim quotes. For example a theme was ‘agreement’ with negative judgements and sub themes included: worthlessness, hopelessness, and negative body image.

### Ethical considerations

This study was approved by the Health Policy and Management and Centre for Global Health Research Ethics Committee in the School of Medicine, Trinity College, Dublin (as it was part of a MSc study for CR) and the Medical Research Council of Zimbabwe as the research was being conducted in Zimbabwe (#2604). Africaid provided information leaflets and consent forms to all the respondents at least seven days in advance of interviews. Written and oral informed consent were obtained from all respondents prior to participation. Confidentiality assurances were given during the consent process. Names and other personal identifiers were removed from transcripts before they were analyzed. All information was stored in an approved cloud account that was double password protected and encrypted for protection. Only the lead researcher had access to the account and transcripts unless recorded permission was given to co-researchers. These procedures met the requirements of General Data Protection Regulation (GDPR) in full. Respondents received US$5 bus fare reimbursements.

## Results

### Respondent characteristics

The mean age of respondents was 22 years. Most of the respondents 12/16 (75%), were CATS and equal number of male and female respondents (Table 1). The majority of respondents, 14/16 (88%), disclosed that they were born with HIV.

**Table 1:** Characteristics of respondents in relation to the Zvandiri program (N = 16).

Respondent ID	Age	Female/Male	CATS or Beneficiary
**01**	24	Female	CATS
**02**	19	Male	CATS
**03**	22	Female	Beneficiary
**04**	22	Male	Beneficiary
**05**	24	Male	CATS
**06**	21	Female	CATS
**07**	18	Female	Beneficiary
**08**	20	Female	Beneficiary
**09**	22	Male	CATS
**10**	24	Male	CATS
**11**	23	Male	CATS
**12**	24	Male	CATS
**13**	24	Female	CATS
**14**	24	Female	CATS
**15**	22	Female	CATS
**16**	21	Male	CATS

Corrigan’s self- stigmatization framework [11] helped to analyse if the respondents actually experienced self-stigma and if so, what self-stigmatizing beliefs they applied onto themselves and the impacts of that. The data indicated that all the respondents experienced self-stigma (the judgements one has towards oneself including beliefs of worthlessness, hopelessness, and self-blame), but it was not felt all the time. Most of the respondents, 10/16 (63%), experienced self-stigma frequently (4 or more times a week) and 6/10 (37%) self-stigmatized only occasionally (once or twice a week). Study findings were grouped into Corrigan’s self- stigmatization framework [11] of the three As: awareness, agreement, and application.

### Awareness

The respondents were very aware of negative community beliefs through cultural norms and personal experiences. Fourteen of the sixteen (88%) respondents disclosed that they were born with HIV and were informed about their status at a young age. Therefore, they reported that they had endured years of discrimination, stigma and prejudiced community beliefs. Even those who were diagnosed much later, two of the sixteen (12%), were old enough to have an understanding of how others perceived PLHIV. They reported hearing gossip and negative comments about PLHIV that made the respondents aware of how their community viewed HIV. Misconceptions about HIV perpetuated the fear and judgements their community had towards PLHIV.

This awareness can come from ‘social stigma’, meaning open acts of discrimination towards people living with HIV. Respondents reported experiencing discrimination from friends, family, classmates and community members.

“*My Aunt treats me bad when she comes here [Harare]*. *She doesn*’*t even want to sit near me*, *talk to me or even shake hands with me*. *It makes me feel worthless*.”

(Participant 6, Female, CATS)

“*Like from that time*, *they didn*’*t want to share like a pen or ruler*. *And other stuff*. *And I was treated like someone who is always sick*”

(Participant 16, Male, CATS)

Awareness did not come exclusively from ‘social stigma’ and several respondents who had kept their status a secret described how they were aware of their community’s negative perception of HIV. They were cognizant of and internalized the mainstream stereotypes about HIV including that PLHIV are unclean, sleeping around, and contagious to touch.

“*In the community it is very difficult because they think HIV is an airborne disease or something*. *That if you play with someone who is HIV positive that you will end up having AIDS or something*.”

(Participant 2, Male, CATS)

### Agreement

The respondents agreed with several beliefs that underpin HIV stigma and stereotypes. Community beliefs typically include that PLHIV are dying, dirty, immoral, worthless or to blame for their condition [33].

“*Yes*, *I sometimes internalize it*, *especially when I am alone*. *I can agree that I am worthless*, *or I can*’*t do certain things*.”

(Respondent 13, Female, CATS)

While most of the respondents did not outwardly say that they agreed with these negative beliefs and stereotypes about PLHIV, their responses demonstrated that they did agree with and experienced negative judgements towards themselves. This may be a result of internalizing negative cultural beliefs over time and agreeing with them. The respondents agreed with beliefs of worthlessness, hopelessness, and negative body image.

#### Worthlessness

When respondents were feeling down about their HIV status, they would describe feeling worthless. They reported that these feelings were more intense when they first found out about their status, causing several to become suicidal. Most respondents (88%) had known their HIV status for at least seven years and described that feelings of worthlessness were more prevalent when they first found out their status. However, the belief of worthlessness was still present and impacting their life.

“*When I am not positive* [towards myself], *I just feel I am not worth it*. *I don*’*t deserve to be on this planet at all*. *Because sometimes my family the way they treat me*, *it makes me feel like I am a burden to them*.”

(Respondent 15, Female, CATS)

These feelings of worthlessness have caused suicidal thoughts for many of the respondents. The respondents reported internalizing the belief that PLHIV did not deserve to live. This demonstrates how HIV-related self-stigma can make the respondents feel worthless and confined by their HIV status.

“*Honestly*, *sometimes I wanted to kill myself*. *I thought why am I living in this world*? *I don*’*t have peace*, *I want peace*. *And I want freedom to do what I want*.”

(Respondent 5, Male, CATS)

“*Initially*, *when I first found out*, *I wanted to commit suicide*.”

(Respondent 7, Female, Beneficiary)

#### Hopelessness

The respondents described days where they felt no hope for themselves or their future. This despair could be overwhelming and limiting, causing stress and sadness. This feeling of hopelessness was raised more frequently when discussing issues pertaining to interpersonal relationships with friends, romantic partners, and community members.

“*Sometimes it makes me feel that there is nothing that I can do which makes me feel very hopeless and worthless and I become very stressed and I start thinking is there ever a time where I can live my life with people who care about me and love me and accept me*.”

(Respondent 13, Female, CATS)

A common community belief is that PLHIV are ‘about to die’. When the respondents agreed with this thought, they felt that their destiny was determined and hopeless. This despair could be overwhelming and limiting, causing them stress and sadness. They reported frequently asking themselves “why me?” They could not rationalize the suffering that their HIV had caused, making them feel hopeless.

“*Why only me*? *Why only me*? *Why am I the only HIV positive person in the house*? *Why*, *why*, *why*, *why*.”

(Participant 1, Female, CATS)

*Negative body image*. Common stereotypes of PLHIV in Zimbabwe and elsewhere are that they are ‘unclean’ and able to spread HIV through touch [7, 34]. Although the respondents knew that these misconceptions were not true, they admitted to internalizing the negative beliefs.

“*Yeah*, *from the beginning I felt that I wasn*’*t worth sitting around people who are HIV negative because I would feel that I was unclean and that I would make them dirty*. *It has gotten better though*.”

(Participant 14, Female, CATS)

These negative perceptions about their body made them feel like they smelled of HIV, should not sit next to people who are HIV negative, and that they could not be loved. This negative body image caused low self-esteem and inhibited the respondents from living their lives freely.

“*When I heard that I was HIV positive*, *it lowered my esteem*, *I started being reserved*. *I started disassociating with people*. *I felt the self-stigma*. *I could walk and think it is written on the wall*; *on my forehead you know*? *I would walk and think it is readable that you are HIV positive*. *That it can be seen*.”

(Respondent 4, Male, Beneficiary)

### Application

After becoming aware of and experiencing HIV-related stigma, the respondents reported internalizing negative judgements expressed by members of their community. The application of these beliefs onto experiences in their lives reinforced their self-stigma. The key themes of self-stigmatizing experiences that became evident in the interviews included disclosure difficulties, self-isolation, and relationship complications.

#### Disclosure

The respondents reported that disclosure was extremely difficult because a lot of trust and time was needed to be able to tell someone about their HIV status. Disclosure is challenging for these respondents because of negative disclosure experiences and their fears of being socially rejected.

“*It is difficult to tell people that you are HIV positive because you will struggle to walk in the community*, *in the neighborhood because people will humiliate you*, *they will shout at you*, *say terrible things about you because you are HIV positive*.”

(Respondent 9, Male, CATS)

The respondents reported worrying that they would lose their friends if they disclosed their status due to the shame of HIV. Therefore, many respondents kept their HIV a secret, even to the detriment of their health by quitting their medication.

“*So*, *I was shy*, *I was embarrassed*. *Like I was still thinking*, *what will they say if they know I am like this*? *Will they even accept me*? *So*, *I just quit my medication which was quite stupid*.”

(Respondent 4, Male, Beneficiary)

“*So*, *my first year in high school*, *I was a boarder*, *and that was quite hard*. *You know you have to take your medication*, *but you live in a dorm with I think 8 girls and you need that privacy to take your medication*. *So*, *I had to quit*.”

(Respondent 3, Female, Beneficiary)

#### Isolation

Isolation and feeling alone was a common theme in the interview responses. Respondents reported being left out by friends and family, feeling like they had no one to talk to, and self-isolating due to their status. This caused self-stigmatization and feelings of loneliness, shame and being silenced.

Respondents endured being left out and separated from other people because of their HIV status and the belief that PLHIV are dirty or dangerous. Misconceptions by others about the virus caused others to exclude the respondents, causing them to feel alone and ‘unclean’.

“*My grandma used to say you guys have to sit there*, *you have to have your own cups*, *you have to have your own things*, *you cannot share with others*. *So*, *it was so hard for me because always I couldn*’*t do what other kids could do because I was always being separated from other kids*.”

(Respondent 14, Female, CATS)

Experiences of stigma caused so much humiliation that the respondents felt deeply ashamed of their status and would isolate themselves. Respondents reported that their shame would hold them back from socializing with others, making them feel alone and without anyone to talk to.

“*It is very painful*, *and I feel sad and sometimes I cry about it*. *And sometimes I feel that I shouldn*’*t even walk in the street or do things*”

(Respondent 13, Female, CATS)

“*It was really hard because I had no one to talk to*. *No one to express how I felt and even my own family*, *they didn*’*t even understand*.”

(Respondent 15, Female, CATS)

Respondents often felt alone because of their HIV status and unable to speak their minds. These feelings were perpetuated through self-stigmatization by believing they were not worthy enough to have a voice.

#### Relationships

Relationships were a significant source of stress and self-doubt for respondents. As adolescents and young adults, all respondents had either experienced being in a relationship or expressed wanting to date. However, this was complicated by fears of disclosure, experiences of rejection, and the status of their partners.

Respondents reported feeling hopeless about finding love and fearful about disclosing to a potential partner. By believing they were unlovable, respondents reported that they would self-sabotage relationships by ending or not engaging in them. These feelings generally got better over time as respondents saw other PLHIV engage in relationships.

“*She said that in the past she didn*’*t use to date anyone because she was HIV positive and so she thought that would result in her being rejected*.”

(Respondent 13, Female, CATS. Translation from Shona by psychologist)

Many of the respondents had painful experiences of being rejected by a partner due to their HIV status. This led to most of the respondents to choose to date only HIV positive people in order to avoid disclosure. However, several respondents (44%) still expressed a desire to date someone not living with HIV.

“*For me it was a big challenge because I was looking for another who was not HIV positive*. *I wanted someone who was HIV negative to date*, *but the issue was disclosure*.”

(Respondent 12, Male, CATS)

### Secondary themes

Secondary themes are reported as important information that came up frequently, but were not as prevalent as the above-described themes. The secondary themes presented are gender differences and coping mechanisms.

#### Gender differences

The respondents were split evenly on whether they thought men and women experienced HIV-related self-stigma differently. While the interview responses demonstrated that men and women have the same self-stigmatizing beliefs, they suggested that the experiences of self-stigma could be more intense for women due to their already marginalized position in society [35]. Half of the respondents felt that experiences of HIV and self-stigma were worse for women.

“*So*, *if a girl*, *if she*’*s positive they will think she is characterless*, *or she has been sleeping around with a lot of boys*. *For the boys*, *if you are HIV positive*, *it doesn*’*t really affect him as much*.”

(Respondent 1, Female, CATS)

“*There is a difference between men and women… even if it*’*s a couple*, *it is the woman who goes to the facility*. *So*, *she is the one who usually is labelled as having HIV or her reputation is usually tarnished in the community because she is HIV positive*. *Whereas men are usually in hiding and not usually associated with infection even though they are HIV positive*”

(Respondent 12, Male, CATS)

#### Coping strategies

The respondents shared how they were able to better manage their HIV and reduce their negative thoughts. These coping strategies included counselling, support groups, hearing shared experiences, and medication adherence. These interventions and support from Africaid helped ease self-stigmatizing beliefs for the respondents.

“*I have had some group counselling with peers*, *with my primary counsellors at my facility*, *and some aunties at Zvandiri [Africaid]*. *They helped me a lot*.”

(Respondent 12, Male, CATS)

“*And so*, *when I started seeing others*, *my peers that were surviving*, *that were going to school*, *that were graduating from university I realized that this [stigmatizing thoughts] wasn*’*t true*, *that there was hope*.”

(Respondent 13, Female, CATS)

## Discussion

Corrigan et al. (2009) defined self-stigmatization as a process in which a person becomes aware of negative stereotypes, finds that they agree with those judgements, and applies the stigmatized beliefs onto themselves [11]. The data was analyzed using this framework and the results of this study clearly report feelings, experiences, and beliefs that are consistent with Corrigan’s definition of self-stigma. Respondents reported the beliefs and feelings of worthlessness, hopelessness, and negative body image. These negative self-perceptions were reinforced by the self-stigmatizing experiences of disclosure difficulties, isolation, and dating complications. Respondents all expressed having frequent (4 or more times a week) or occasional (once or twice a week) negative feelings towards themselves because of their HIV status.

All respondents were aware of the harmful stereotypes ascribed to PLHIV. They heard negative comments, witnessed intolerance, and endured personal humiliation as a direct result of their HIV status. There are several societal beliefs and cultural factors that made the respondents aware of HIV stigma. The negative association between HIV and the lesbian, gay, bisexual, transgender and queer/questioning (LGBTQ) community and the fact that acts of homosexuality are illegal in Zimbabwe adds additional stress and fear to PLHIV when disclosing their HIV status to friends, family, or partners-regardless of their sexuality [23]. Despite most of the respondents being born with HIV, they still felt blamed for living with HIV and were associated with ‘immoral’ behaviors such as multiple sexual partners or drug use. Additionally, the respondents reported that being called in Shona the equivalent of “dead one” or “plagued one” led them to believe that their lives were hopeless. These stereotypes perpetuate negative conceptions of HIV and reinforced adverse cultural norms that the respondents internalized.

Not only were the respondents aware of the societal stereotypes of PLHIV, but they also agreed with many of the underlying beliefs associated with them. These negative self-perceptions included beliefs of worthlessness, hopelessness, and negative body image. These beliefs caused the respondents to feel limited in their agency to live their lives the way they wanted. However, the respondents explained that these beliefs were more intense when they first found out their diagnosis. Self-stigmatizing thoughts can decrease over time with proper interventions and are not a static experience. Most of the respondents had known their status for at least seven years. Therefore, it would be interesting to investigate further how self-stigmatizing beliefs vary with length of time knowing one’s HIV status and mode of transmission.

Believing that their lives were worthless and hopeless caused many of the respondents to experience depression or suicidal thoughts. This led to emotional distress which resulted in poor adherence to anti-retroviral regimens. When they believed their lives were no longer worthwhile or feared they would be severely judged, the respondents decided to stop taking their medication. Without medication, the respondents experienced an increased viral count, causing extreme weight loss, and increased susceptibility to AIDS-related illnesses [36]. Additionally, these beliefs deeply impacted their ability to easily connect with friends or family, inhibiting them from a support network.

Interestingly, while the respondents felt hopeless in their personal relationships and ability to be accepted by their community, they generally did not feel hopeless when talking about their career. Corrigan et al. (2009) explained that self-stigma can deter people from going after their dreams and careers due to feelings of worthlessness and hopelessness. However, in this study, most of the respondents explained that attending counselling sessions and seeing other PLHIV who had thriving careers were sources of inspiration and encouragement. Research has shown that support groups are a useful resource for facilitating self-acceptance and restoring the confidence that may be lost once one is diagnosed with a highly stigmatized infection such as HIV [20, 37]. This supports the transformative effects of support groups, however, these groups must also address self-stigmatizing beliefs that respondents’ experience.

The respondents each spoke of several experiences in which they were aware of negative self-judgments including disclosure of their HIV status and dating with interpersonal relationships of paramount importance to this age group. As young adults, peer acceptance is extremely important for support and determining self-worth [38]. It is an age where young people are trying to find their individuality, yet also desire community acceptance [38]. Study respondents reported that living with HIV created a tension between the need to be themselves while also maintaining social and community acceptance.

Respondents felt that by disclosing their HIV status, they would not be accepted by their community. This realization caused stress and fear in trying to decide whether to disclose to friends or family. Several of the respondents were still in school, a place that commonly facilitates gossip and social exclusion towards PLHIV [39]. This caused most of the respondents to avoid disclosing their HIV status to anyone. However, the burden of this secret made many of the respondents feel shameful and stressed, leading them to avoid engagements with friends, skip doctor’s appointments, and stop taking their medication. This caused both physical and emotional health problems for the respondents and likely explains the high rates of virological failure seen in this group [22, 27].

By not disclosing or talking to anyone about their HIV, the respondents described an intense loneliness and isolation. It often was not that the respondents had literally no one to talk to. Instead, their self-stigmatizing beliefs made them feel so shameful and worthless that they were unable to initiate needed conversations for support. These feelings caused the respondents to self-isolate and pull away from friends, family, and social gatherings. Studies have shown that PLHIV silence themselves to avoid conflict, judgement, and to conform to societal standards [40, 41]. However, the respondents reported that the burden of living with the secret of their HIV status was extremely overwhelming to handle alone [41].

The respondents were 18–24 years old, an age range where dating and relationships are often important and a priority [42]. This was clear because the topic was discussed at length in the interviews. However, as important as relationships were to them, dating also caused stress and complications for the respondents. Their self-stigmatizing beliefs made them feel hopeless to find a partner that would ever accept them. This was immensely saddening and discouraging for respondents who wished to start a family. Past rejection, abuse, and stigma caused low self-confidence and fear when trying to date or disclose to a partner. Studies have shown that anxiety and shame before or during disclosure in relationships are common for young adults with HIV [43, 44]. Therefore, when the respondents self-stigmatized, their shame normally caused them to end the relationship before they had to face disclosure. Beliefs of worthlessness and shame were most closely tied to dating and interpersonal relationships, indicating the importance of this topic.

Respondents who did decide to date, often found it easiest to date someone who also had HIV. This helped them to avoid the disclosure process and feel less ashamed. However, several of the respondents (44%) admitted to wishing they were dating someone who was HIV negative. This suggests deep rooted judgements towards themselves and HIV, because they could not even fully accept someone who shared in their same condition. There is little literature on this phenomenon with PLHIV and dating, however, this stigmatization towards one’s own group has been seen in other marginalized communities, such as those in the LGBTQ community and people with mental illnesses [45, 46]. These studies linked that those who experienced self-stigma could also stigmatize others within their group due to the negative beliefs they hold about their group identity [45, 46]. Therefore, further research should investigate the complicated experiences of self-stigma and dating in this age group due to its importance and influence on the respondents.

This study had a gender balance of 50% men and 50% women. Both sexes reported very similar beliefs and experiences of HIV-related self-stigma. They shared the same feelings of worthlessness, hopelessness and negative body image when dating, disclosing their status and feeling isolated. Yet, both men and women in the study felt that society was harsher and more discriminatory towards HIV positive women than men. Half of the women (n = 4) discussed the possibility that the increased prejudice they experienced as women caused them to self-stigmatize more often and more intensely than men. A study undertaken by Duffy (2005) concluded that women with HIV were more discriminated and more frequently blamed for spreading HIV in Zimbabwe than men [47]. Reidpath et al. (2005) explain that by holding two stigmatized identities in society, such as being woman and a person living with HIV, one can experience layered or additive stigma, which can increase the frequency and intensity of self-stigma [5]. This was only reported amongst half of women and we did not have quantitative scales to measure self-stigma intensity. Therefore, gender differences should be further explored as a potentially impactful aspect of self-stigma with a larger group of respondents.

Taking these findings into consideration for future interventions is critical in addressing HIV-related stigma. Study respondents were already receiving counselling and engaged in support groups. Additionally, most of the respondents were CATS which meant that they had training and on-going supervision in the provision of counselling which has been found to alleviate feelings of hopelessness, worthlessness, feeling unloved, isolation, depression and suicidality. That all of the respondents still reported self-stigmatizing beliefs despite the support highlights the need to specifically address self-stigma within the Zvandiri program (now in nine countries) and to remain mindful of CATS’ own support needs. Previous research suggested a reduction in stigma among ALHIV supported by CATS [48] and further suggested that engagement with CATS improves self-acceptance, self-worth and confidence [20, 49, 50]. Innovative strategies to both enhance and sustain this transformative effect should continue to be explored.

Importantly, our study suggests that context is hugely key in the way that HIV is understood and then impacts the way that PLHIV live with, and experience, HIV. This is especially salient in the colloquial references to PLHIV which condemn these individuals some kind of “half-life”. As such, interventions that support PLHIV in general and AYALHIV in particular, need to be context-relevant if they are going to impact on self-stigma at all. Additionally, interventions should be systemically applied (i.e. policy and politics need to change attitudes at societal, community, household levels while HIV services build individual resilience in the meantime). It will be important to work across the entire socioecological framework since individuals are embedded within larger social systems and multiple levels of influence not only exist but are also interactive and reinforcing [51].

Of note, our team is currently piloting a peer-led intervention to support AYALHIV in Zimbabwe on the journey from self-stigma to self-worth using a combination of inquiry-based stress reduction, music, art and creative expression (all have been previously run separately and successfully by the team) [13, 21, 52, 53]. Addressing and reducing self-stigma will likely enhance AYALHIV’s confidence to take medication, disclose their status safely, and take appropriate healthcare measures [13]. The spiral effect will be the much-desired improvement in AYALHIV’s clinical and psychosocial outcomes [15, 22, 30].

### Strengths and limitations

As a qualitative study, this research reports in-depth accounts and analysis of the lived experiences of HIV self-stigma. The interviews helped create a rich description of the thoughts and beliefs that PLHIV experience about themselves. Since there are very few studies on HIV self-stigma, this study makes a valuable contribution to the understanding of this important issue and provides an opportunity for Africaid’s Zvandiri programme and other HIV support interventions to specifically address the impacts of self-stigma.

There were some limitations to this study. CR, a non-Zimbabwean, led the interviews in English which may have made it difficult for respondents to connect with the researcher, in addition to creating a power imbalance [54]. A few of the respondents chose to respond to certain questions in Shona, which was translated live by the Zimbabwean psychologist (VM) present. Live translations have the potential to not capture all that was originally said or to be accidentally misinterpreted [55].

Due to the COVID-19 pandemic, availability of respondents was limited; therefore, twelve of the sixteen respondents were CATS. CATS have been trained to help young people living with HIV and teach them coping mechanisms, so this type of training and knowledge about HIV may have had an impact on their responses. The CATS training and increased access to HIV resources could alter their beliefs and therefore potentially report lower levels of self-stigma than other PLHIV. This limits the generalizability of the results of the study because most young PLHIV do not have as easy access to HIV resources as the CATS do. However, the fact that CATS still reported HIV-related self-stigma despite their training and experience as peer counsellors indicates that self-stigma is still a pervasive and unaddressed issue.

COVID-19 also caused the interviews to be online over video call rather than in person. In person interviews are generally regarded as the preferred method for talking about sensitive issues, such as HIV, so that the respondent feels more comfortable [56]. Therefore, doing these interviews online could have inhibited the respondents from opening up as much as they may have wanted to about their HIV experiences. Despite these limitations, we still hope that we managed to elicit important self-stigma information that will inform stigma-reduction interventions.

## Conclusions

This study explored the beliefs, experiences, and impacts of HIV-related self-stigma amongst young adults living with HIV in Harare. The results of this study are critical to our understanding of how adolescents and young adults in Harare, Zimbabwe experience HIV-related self-stigma and will inform Africaid’s Zvandiri programme and other HIV support intervention’s efforts to address the impacts of self-stigma. Coping strategies for self-stigma that were helpful for the respondents should be incorporated into existing and future HIV interventions by addressing self-stigmatizing thoughts in counselling, support groups, inquiry-based stress reduction, art, meditation, and mindfulness techniques. This will help young adults struggling with HIV-related self-stigma to recognize their self-judgements and constructively change the way they view themselves. Additionally, engaging more young people in interventions that challenge self-stigma can help PLHIV build a community and support network that is clearly needed amongst this age group. By promoting awareness of self-stigma, PLHIV may be empowered to challenge the perspectives of others, attend social gatherings, talk to their partner about their HIV status, and proactively engage with HIV support services.

## Supporting information

S1 File Interview guide. (DOCX)
