# Supplementary material for: Exploring the beliefs, experiences and impacts of HIV-related self-stigma amongst adolescents and young adults living with HIV in Harare, Zimbabwe: A qualitative study
Source: PLoS One. 2022 May 18;17(5):e0268498. doi: 10.1371/journal.pone.0268498 (PMC9116620; doi:10.1371/journal.pone.0268498)
Supplement: S1 File — (DOCX) [file pone.0268498.s001.docx]

Interview Guide

*(Note to interviewer: Questions in bullet form are meant to be probes. They do not have to be asked as they appear here. Rather, phrase and order questions according to the flow of interview).*

Introduction/Demographics

- Tell me a little bit about yourself and where you grew up?
  - Age
  - Married, unmarried, partner?
  - Work or school?
- How long have you been involved with Africaid?
- How would you describe yourself to other people?

*I am now going to ask you some questions about your experiences living with HIV*

General questions about living with HIV

- How long have you known your HIV status?
- Describe what it is like living with HIV
  - How has your HIV status effected your daily life?
- Have you disclosed to your friends and family about your status?
  - Why or why not?
  - If no: Do you normally try to keep your HIV status a secret from others?
    - Why or why not?
  - What has been your experience when you either tell people or they find out you are living with HIV?

Awareness

- How do you think society perceives people living with HIV?
  - How do those perceptions make you feel?
  - Are these perceptions different for men and women?
- What are some of the challenges you or other young adults face living with HIV?
  - Do you think that young adults face challenges around HIV that are different than people living with HIV who are older or younger?

Agreement

- How does your HIV status make you feel?
  - Do you think your age or gender influence those feelings?
- Did finding out that you are living with HIV change your perception of yourself at all?
  - If yes: How so?
  - Probe: Has that perception changed over time? Child versus now adult?

Application

- We are going to try an exercise that you can either write down or think about. I will give you a few minutes to complete the sentence: I am HIV positive and that means that…
- How do these feeling and beliefs about yourself influence how you make life decisions?
- What are your current hopes and goals for the future?
  - Has your feeling towards yourself or your HIV status impacted these goals?
- How has living with HIV impacted your friendships or relationships?

Recommendations

- What do you think could be done to help people cope with their HIV status better or reduce self-stigma?

Conclusion

- Is there anything else you wish to tell me that we have not covered?
